# Supplementary material for: Adaptive and degenerative evolution of the S-Phase Kinase-Associated Protein 1-Like family in Arabidopsis thaliana
Source: PeerJ. 2019 Apr 12;7:e6740. doi: 10.7717/peerj.6740 (PMC6463862; doi:10.7717/peerj.6740)
Supplement: Supplemental Information 1 [file peerj-07-6740-s001.docx]

**Table S1.** OrthoMCL groups of Skp1 proteins in *Ath*, *Aly*, and *Aha* species

| Groups | *Ath* | *Aly* | *Aha* |  |
| --- | --- | --- | --- | --- |
| OrthoMCL1 | ASK7,ASK8,ASK9,ASK10 | AlySkp09 | AhaSkp05 |  |
| OrthoMCL2 | ASK3,ASK4 | AlySkp04 | AhaSkp03 |  |
| OrthoMCL3 | ASK11,ASK12 | AlySkp06 |  |  |
| OrthoMCL4 | ASK1 | AlySkp01 | AhaSkp01 |  |
| OrthoMCL5 | ASK19 | AlySkp03 | AhaSkp02 |  |
| OrthoMCL6 | ASK2 | AlySkp05 | AhaSkp04 |  |
| OrthoMCL7 | ASK13 | AlySkp13 | AhaSkp06 |  |
| OrthoMCL8 | ASK18 | AlySkp08 | AhaSkp07 |  |
| OrthoMCL9 | ASK16 | AlySkp12 | AhaSkp08 |  |
| OrthoMCL10 | ASK5 | AlySkp10 | AhaSkp09 |  |
| OrthoMCL11 | ASK14 | AlySkp15 | AhaSkp10 |  |
| OrthoMCL12 | ASK6 | AlySkp02 | AhaSkp11 |  |
| OrthoMCL13 | ASK17 | AlySkp07 |  |  |
