# Supplementary material for: Adaptive and degenerative evolution of the S-Phase Kinase-Associated Protein 1-Like family in Arabidopsis thaliana
Source: PeerJ. 2019 Apr 12;7:e6740. doi: 10.7717/peerj.6740 (PMC6463862; doi:10.7717/peerj.6740)
Supplement: Supplemental Information 2 [file peerj-07-6740-s002.docx]

**Table S2.** Gene identifications of 15 known F-box proteins selected for testing the differential interactions with ASK1 and ASK2

| Gene Name | Gene Identification | |
| --- | --- | --- |
|  | AGI | (Hua et al. 2011) |
| *FBP7* | *AT1G21760* | *At_F0001* |
| *UFO* | *AT1G30950* | *At_F0267* |
| *SKIP4* | *AT3G61350* | *At_F0437* |
| *PP2-A12* | *AT1G12710* | *At_F0514* |
| *CFB* | *AT3G44326* | *At_F0515* |
| *FBL17* | *AT3G54650* | *At_F0563* |
| *JMJ22* | *AT5G06550* | *At_F0649* |
| *SKIP16* | *AT1G06110* | *At_F0664* |
| *FBS4* | *AT4G35930* | *At_F0709* |
| *MEE11* | *AT2G01620* | *At_F0710* |
| *LCR* | *AT1G27340* | *At_F0313* |
| *KMD3* | *AT2G44130* | *At_F0354* |
| *DIF* | *AT1G30090* | *At_F0466* |
| *CFK1* | *AT5G42350* | *At_F0220* |
| *KFB* | *AT1G23390* | *At_F0500* |

#AGI: Arabidopsis genome identification

**References**

Hua Z, Zou C, Shiu SH, and Vierstra RD. 2011. Phylogenetic comparison of F-Box (FBX) gene superfamily within the plant kingdom reveals divergent evolutionary histories indicative of genomic drift. *PLoS One* 6:e16219. 10.1371/journal.pone.0016219
