# Supplementary material for: Adaptive and degenerative evolution of the S-Phase Kinase-Associated Protein 1-Like family in Arabidopsis thaliana
Source: PeerJ. 2019 Apr 12;7:e6740. doi: 10.7717/peerj.6740 (PMC6463862; doi:10.7717/peerj.6740)
Supplement: Supplemental Information 4 [file peerj-07-6740-s004.pdf]

```
            1
ASK18       SSEEIVSA GESSEIEE AVASLTMS SNKILLTS SDGESFEI DEAVARKF LIIVHMME DNCAGEAI PLENVTGD ILSKIIEY AKMHVNEP SEEEAKKN
AlySkp08    SSSKKSSD GKSSEIEE AVASLTMP FNKILLTS SDGESFEI DEAVARKF QIIAHMIE DDCAGKAI PIDNVTGD ILSKIIEY AKKHVVEP -DEEAKKK
AhaSkp07    SPSKKSSA GKSSEIEE AVASLTMS SNKILLTS SDGESFEI DEAVARKF QIIAHMIE DDCAGKAI PIDNVTGD ILSKIIEY AKKHVVEP -DEEAKKK
ASK19       -------- -------- ------MS SKKIVLTS SDGESFKV EEVVARKL QIVGHIIE DDCATNKI PIPNVTGE ILAKVIEY CKKHVED- -DDDKKEK
ASK15       -------- -------- ------MS SNKIVLTS SDGESFQV EEVVARKL QIVKHLLE DDCVINEI PLQNVTGN ILSIVLEY CKKHVDDV VDDEAKQN
AhaSkp02    -------- -------- ------MS SNKIVLTS SDGESFQV EEVVARKL QIVGHMIE DDCVIKSI PLQNVTGK TLSMVLEY CKKHVDDV VDDEAKKK
AlySkp03    -------- -------- ------MS SNMIVLTS SDGESFQV EEVVARKL QIVGHMLE DDCVINAI PLQNVTGK TLSMVLEY CKKHVDDV VADEAKKK
ASK16       -------- -------- ------MS SNKIVLTS SDDESFEV EEAVARKL KVIAHMID DDCADKAI PLENVTGN ILALVIEY CKKHVLDD VDDEAKNE
ASK17       -------- -------- ------MS SKKIVLTS SDDECFEI DEAVARKM QMVAHMID DDCADKAI RLQNVTGK ILAIIIEY CKKHVDDV ---EAKNE
AlySkp07    -------- -------- ------MS SNKIVLTS SDDESFEV DEAVARKS QIIAHMID EDCADKAI LLQNVTGK ILAIIIEY CKKHVDD- -VDAKNEL
AhaSkp08    -------- -------- ------MS SNKIVLTS SDDESFEV DEAVARKL QIIAHMID DDCANKAI PLQNVTGK ILAMVIEY CKKHVNDV DSDEAKNE
AlySkp12    -------- -------- ------MS SNKIVLTS SDDESFEV DEAVARKL QIIAHMID DDCADKAI PLQNVTGK ILAVVIEY CKKHVNDV DDSEAKKE
ASK14       -------- -------- ------MS SNKIVLSS SDGESFEV EEAVARKL KIVEHMIE DDCVVTEV PLQNVTGK ILSIVVEY CKKHVVD- ---EESDE
AlySkp15    -------- -------- ------MS SNKIVLTS SDGESFEV EEVVARKL QIVGHMLE DDCVINEI PLQNVTGD ILSMVIEY CKTHVDE- -EEEAQTK
AhaSkp10    -------- -------- ------MS SNKIVLTS SDGESFEV EEVVARKL QIVGHMLE DDCVINEI PLQNVTGD ILSMVIEY CKEHVDE- -EEEAQTK
ASK6        -------- -------- -------- -------- -------- -------- MMIKGMAE DDCADNGI PLPNVTSK ILLLVIEY CKKHVVE- -S--KEED
AlySkp02    -------- -------- -------- -MVILFRS T------- KRAVAFQS AMIKGMDE DKCADNGI PLPNVTSK ILLLVIEY CKKHVVE- -N-EEEEY
AhaSkp11    -------- -------- ------MA KKIIVLTS SDGDSFQI DEAVAFQS AMIKGMDE DKCADNGI PLPNVTSK ILLLVIEY CKKHVVE- -S-NEEED
ASK5        -------- -------- -------M STKIMLKS SDGKSFEI DEDVARKS IAINHMVE DGCATDVI PLRNVTSK ILKIVIDY CEKHVKS- ---KEEED
AhaSkp09    -------- -------- ------MS KKMIVLKS SDGKSFEV DEAVALLS KTITQMSE VECAANEI KLLNVTSK ILKIVIAY CKKHVES- ---NEEED
AlySkp10    -------- -------- ------MS KKMIVLKS SDGKSFEV DEAVARKS VTINNMAE DECADNGI PLPNVTSK ILKIVIAY CKKHVES- ---NEEED
AlySkp14    -------- -------- -------- -------- -------- -------- -------- -------- -------- -------- -------- --------
AlySkp16    -------- -------- -------- --MISLTS SDGQTFEI KEDAARQC QIINHMIE DDCADREI PLPNVTGK ILAMVLEY CKKHHVD- -DASTDED
AhaSkp05    -------- -------- ------MS TNKITLKS SDGHSFDV EKDAACQS QIIAGMIE DDCTDNGI LLA-VTSK ILSMVVEY IKKHHVD- -AASTDED
AlySkp09    -------- -------- ------MS AKIIKLKS SDGESFEI KEEAARQS QTIFHLID DDCTDKEI PVPNVTGK ILSMVVEY LNKHHVG- -DASTDED
ASK10       -------- -------- ------MS TKKIILKS SDGHSFEV EEEAACQC QTIAHMSE DDCTDNGI PLPEVTGK ILEMVIEY CNKHHVD- -AACSDED
ASK9        -------- -------- ------MS TKKIILKS SDGHSFEV EEEAARQC QIIAHMSE NDCTDNGI PLPNVTGK ILAMVIEY CNKHHVD- -AACSDDD
ASK7        -------- -------- ------MS TKKIMLKS SDGKMFEI EEETARQC QTIAHMIE AECTDNVI PVSNVTSE ILEMVIEY CNKHHVD- -AACSDED
ASK8        -------- -------- ------MS TKKIMLKS SEGKTFEI EEETARQC QTIAHMIE AECTDNVI LVLKMTSE ILEMVIEY CNKHHVD- -AACSDDD
ASK13       -------- -------- -------M SKMVMLLS SDGESFQV EEAVAVQS QTIAHMIE DDCVANGV PIANVTGV ILSKVIEY CKKHVVS- -DSESKDE
AhaSkp06    -------- -------- -------- -------- -------- -------- -----MVE DDCVGDEI PILNVTGA TLSKVIEY CKKHVVA- -ESESKDE
AlySkp13    -------- -------- -------M SKMVMLQS SDGESFQV EEAVAVQS QTIAHMVE DDCVGDGI PVSNVTGA TLSKVIEY CKKHVVA- -AEEEWDE
AlySkp17    -------- -------- ------MA SNKIILTS SNGESFEI DEAVAVES LTIKHMIE DDCAGNGI PLPSVIGG ILAKVIEC CKKHVET- -AATENKE
AlySkp11    -------- -------- ------MA AKKIILKS SDGESFEI DEAVAVES QTIKHMIE DDCADNGI PLPNVTGA ILAKVIEY CKKHVEA- -AATENDE
AlySkp04    -------- -------- ------MA AKKIILKS SDGESFEV DEAVAVES QTIKHMIE DDCADNGI PLPNVTGA ILAKVIEY CKKHVEA- -AATENDE
AhaSkp03    -------- -------- ------MA AKKIILKS SDGESFEV DEAVAVKS QTIKHMIE DDCADNGI PLPNVTGA ILAKVVEY CKKHVEA- -AATENDE
ASK3        -------- -------- ----MAET KKMIILKS SDGESFEV EEAVAVES QTIKHMIE DDCVDNGI PLPNVTGA ILAKVIEY CKKHVEA- -AATENHE
ASK4        -------- -------- ----MAET KKMIILKS SDGESFEI EEAVAVKS QTIKHMIE DDCADNGI PLPNVTGA ILAKVIEY CKKHVEA- -AAAENDE
AlySkp06    -------- -------- ------MS STMIVLMS SDGQSFEV EEAVAIQS QTIAHMVE DDCAANGI PLANVTSK TLAKVIEY CKKHHVD- -EAISEDE
ASK12       -------- -------- ------MS SKMIVLMS SDGQSFEV EEAVAIQS QTIAHMVE DDCVADGI PLANVESK ILVKVIEY CKKYHVD- -EAISEED
ASK11       -------- -------- ------MS SKMIVLMS SDGQSFEV EEAVAIQS QTIAHMVE DDCVADGI PLANVESK ILVKVIEY CKKHHVD- -EAISEED
ASK2        -------- -------- -----MST VRKITLKS SDGENFEI DEAVALES QTIKHMIE DDCTDNGI PLPNVTSK ILSKVIEY CKRHVEA- -AESSDED
AhaSkp04    -------- -------- -----MST VRKITLKS SDGENFEI DEAVALES QTIKHMIE DDCADNGI PLPNVTSK ILSKVIEY CKKHVEA- -AASSDED
AlySkp05    -------- -------- -----MST VRKITLKS SDGENFEI DEAVALES QTIKHMIE DDCADNGI PLPNVTSK ILSKVIEY CKKHVEA- -AASSDED
ASK1        -------- -------- ------MS AKKIVLKS SDGESFEV EEAVALES QTIAHMVE DDCVDNGV PLPNVTSK ILAKVIEY CKRHVEA- -AATSDDD
AhaSkp01    -------- -------- ------MS SKKIVLKS SDGESFEV EEAVALES QTIAHMVE DDCVDNGV PLPNVTSK ILAKVIEY CKKHVEV- -AATSDED
AlySkp01    -------- -------- ------MS SKKIVLKS SDGESFEV EEAVALES QTIAHMVE DDCVDNGV PLPNVTSK ILAKVIEY CKKHVEV- -AATSDED
```

```
          97
ASK18     LDSWDAKF MEKLDLET IFKIILAA NYLNFEGL LGFASQTV -----ADY IKDKTPEE VREIFNIE NDFTPEEE EE-IRKEN AWTFNE-- -------
AlySkp08  LDSWDAKF VEKLDLET IFKIILAA NYLNFEGL LGFASQTV -----ADY IKDKTPEE VREIFNIE NDFTPEEE EA-IRKEN AWTFNG-- -------
AhaSkp07  LDSWDAKF VEKLDLET IFKIILAA NYLNFEGL LGFASQTV -----ADY IKDKTPEE VREIFNIE NDFTPEEE EE-IRKEN AWTFNE-- -------
ASK19     LNEWDAKF MKDFDIKT IFDIILAA NYLNVQGL FDLCSKTI -----ADY IKDMTPEE VRELFNIE NDFTPEEE EA-IRNEN AWTFEQDG KQQVPKP
ASK15     LDAWDAEF MKNIDMET IFKLILAA NYLNVEGL LGLTCQTV -----ADY IKDKTPEE VRELFNIE NDFTHEEE EEAIRKEN AWAFEADT KHEDPKP
AhaSkp02  LDAWDAEF MKDLNMET IFNIILAA NYLNVKGL LDLTCQTV -----ADH IKDKTPEE VRVIFNIE NDFTPEEE EA-IRKEN AWTFGADS KTEDPKP
AlySkp03  LDAWDAKF MKDLNTET IFSIILAA NYLNVKGL LDLTSQTV -----ADY IKDMTPEE VRELFNIE NDFTPEEE EA-IRKEN AWTFEAAS AQEVPKP
ASK16     LRTWDAEF MKEFDMET VMKLILAV NYLNVQDL LGLTCQTV -----ADH MKDMSPEE VRELFNIE NDYTPEEE DA-IRKEN AWAFEDLK -------
ASK17     FVTWDAEF VKNIDMDT LFKLLDAA DYLIVIGL KNLIAQAI -----ADY TADKTVNE IRELFNIE NDYTPEEE EE-LRKKN EWAFN--- -------
AlySkp07  VMTWDAEF MKNIDMET VFKLLNAA DYLNVKGL LDLTSNTI -----ADY IKDKKVEE IREIFNIE NDYTPEEE EE-LRKQK AWAFKDQN -------
AhaSkp08  LVAWDAEF MKNIDMDT IFNLLLAA NYLNVKGL LDLTSQTI -----ADY IKVMTPEK VREFFNIE NDFTPEEE EE-IRKEN EWAFEDLN -------
AlySkp12  LVTWDAEF MKDIDMET MFQLLLAA NYLNVKSL LDLTSQTI -----ADY IKDKTPQE VREIFNIE NDFTPEEE EE-IRKEN EWAFEDLN -------
ASK14     FKTWDEEF MKKFDQPT VFQLLLAA NYLNIKGL LDLSAQTV -----ADR IKDKTPEE IREIFNIE NDFTPEEE AA-VRKEN AWAFE--- -------
AlySkp15  LKTWDEEF MKKFDIKT LLQIILAA NYLNVKGL LDLVSQTI -----ADT IKDYTPEQ IREVFGVE NDYTEEEE AE-VRKEN AWAFDEAD TPKP---
AhaSkp10  LKTWDDEF TKRFDLQT LLKIILAA NYLNVKGL LDLVSQTV -----ADS IKDYTPEK IREVFGIQ NDYTEEEE AE-VRKEN AWAFE--- -------
ASK6      LKKWDAEF MKKMEQSI LFDVMMAA NYLNIQSL LDLTFSNC -------- -------- -R------ -------- -------- -------- -------
AlySkp02  LKKWDTEF MKKMEQSI VFDVMMAA NYLNIQSL IDLTCKTV -----ADF LSGKTPEE IRAYFKIE NDFTPEEE AE-ILREN QWAFE--- -------
AhaSkp11  LKKWDTEF MKKMEQSI VFDVMMAA NYLNIQSL IDLTCQTV -----ADL LSGKTPEE IR------ ------EE AK-IRMEN QWAFE--- -------
ASK5      LKEWDADF MKTIETTI LFDVMMAA NYLNIQSL LDLTCKTV SDLLQADL LSGKTPDE IRAHFNIE NDLTAEEV AK-IREEN QWAFQ--- -------
AhaSkp09  LMEWDADF MKKIEPSI LFDVMMAA NYLCTQTL LDLTCQTV AALLQADL LSGKTPAE IRARFNIE NDLTPAEV AE-IRKEN QWAFE--- -------
AlySkp10  LKEWDADF MKKIEPSI LFDVMIAA NYLNIPSL LDLTCQTV AALLQADL LSGKTPAE IRTRFNIE NDLTPAEV AE-IRKEN QWAFE--- -------
AlySkp14  ---MGRKV ME-KDQLT LFDLINAA SYLDIQSL LDLACQTA -----SDM SKAKTLDQ TREFFNIE NDFTPEEE KA-VLKDY QKAFE--- -------
AlySkp16  LKKWDEKF ME-KDQLT LFDLINAA SYLDIQSL LDLACQTA -----SDM SKAKTLDQ TREFFNIE NDFTPEEE KA-VLKDY QKAFE--- -------
AhaSkp05  LKKWDAHF MQ-IDQST IFDLIMVA NHLEIKSL IDLTCQTV -----ADM IKEETPKQ IRERFNIE NDFTPEEE KA-VLKNY QKAFE--- -------
AlySkp09  LKKWDAEF MQ-IDQST IFDLIMAA NHLNIKSL TDLTCQTV -----ADM IKEETPKQ IRQRFNIE NDFTPEEE KA-VLKNY QKAFE--- -------
ASK10     LKKWDKEF ME-KYQST IFDLIMAA NYLNIKSL LDLACQTV -----ADM IKDNTVEH TRKFFNIE NDYTHEEE EA-VRREN QWGFE--- -------
ASK9      LKKWDKEF ME-KDTST IFDLIKAA NYLNIKSL FDLACQTV -----AEI IKGNTPEQ IREFFNIE NDLTPEEE AA-IRREN KWAFE--- -------
ASK7      LKKWDKEF ME-KDQYT IFHLMNAA YDLHIKSL LALAYQTV -----ADM V------- -------- -------- -----NDN KWAFE--- -------
ASK8      LEKWDKEF ME-KDKST IFALTNAA NFLNNKSL LHLAGQTV -----ADM IKGNTPKQ MREFFNIE NDLTPEEE AA-IRREN KWAFE--- -------
ASK13     LKKWDAEF MKALEQST LFDVMLAA NYLNIKDL LDLGCQTV -----ADM ITGKKPDE IRALLGIE NDFTPEEE EE-IRKEN QWAFE--- -------
AhaSkp06  LKKWDAEF MKAMEQST LFHVILAA NYLNIKDL LDLGCQTV -----ADT ITDKNPDE IRALLGIR NDFTPVEE EE-IRQQN QWAFE--- -------
AlySkp13  LKKWDAEF MKAMEQST LFHVILAA NYLNIKDL FDLGCQTV -----ADT ITDKNTDE IRALFGIR NDFTPEEE EE-IRQQN QWAFE--- -------
AlySkp17  LKAWDADF VQ-VDQPI LFDTILVA NYLNNSGL LDLTCKTV -----DDM MREKTPEE MRAHFNIK NDYSAEEE EKPYKDQN VKNFKN-- -------
AlySkp11  LKAWDNDF VK-VDQPT LFDLILAA NYLNISGL LDLTCKAV -----ADM MRGKTPEQ MREHFNIK NDYTPEEE EE-VRNEN KWAFE--- -------
AlySkp04  LKAWDNDF VK-VDQPT LFDLILAA NYLNISGL LDLTCKAV -----ADM MRGKTPEQ MREHFNIK NDYTPEEE AE-VRNEN KWAFE--- -------
AhaSkp03  LKAWDNDF VK-VDQPT LFDLILAA NYLNISGL LDLTCKAV -----ADM MRGKTPEQ MREHFNIK NDYTPEEE AE-VRNEN KWAFE--- -------
ASK3      LKTWDNDF VK-VDHPT LFDLLRAA NYLNISGL LDLTCKAV -----ADQ MRGKTPAQ MREHFNIK NDYTPEEE AE-VRNEN RWAFE--- -------
ASK4      LKNWDSEF VK-VDQPT LFDLILAA NYLNIGGL LDLTCKAV -----ADQ MRGKTPEQ MRAHFNIK NDYTPEEE AE-VRNEN KWAFE--- -------
AlySkp06  LKKWDTEF ME-TDQST IFDLILAA NYLNIKSL LDLTCQTI -----ADM IKGKNPEE IRTLFNIK NDFTPEEE EE-VRREN QWAFE--- -------
ASK12     LNKWDEKF MD-LEQST IFELILAA NYLNIKSL FDLTCQTV -----ADM IKGKTPEE IRSTFNIE NDFTPEEE EA-VRKEN QWAFE--- -------
ASK11     LNNWDEKF MD-LEQST IFELILAA NYLNIKSL LDLTCQTV -----ADM IKGKTPEE IRSTFNIE NDFTPEEE EA-VRKEN QWAFE--- -------
ASK2      LKTWDSEF IK-VDQGT LFDLILAA NYLNIKGL LDLTCQTV -----ADM IKGKTPEE IRKTFNIK NDFTPEEE EE-VRREN QWAFE--- -------
AhaSkp04  LKTWDSEF IK-VDQGT LFDLILAA NYLNIKGL LDLTCQTV -----ADM IKGKTPEE IRKTFNIK NDFTPEEE EE-VRREN QWAFE--- -------
AlySkp05  LKTWDSEF IK-VDQGT LFDLILAA NYLNIKGL LDLTCQTV -----ADM IKGKTPEE IRKTFNIK NDFTPEEE EE-VRREN QWAFE--- -------
ASK1      LKAWDADF MK-IDQAT LFELILAA NYLNIKNL LDLTCQTV -----ADM IKGKTPEE IRTTFNIK NDFTPEEE EE-VRREN QWAFE--- -------
AhaSkp01  LKAWDTEF MK-IDQAT LFELILAA NYLNIKNL LDLTCQTV -----ADM IKGKTPEE IRTTFNIK NDFTPEEE EE-VRREN QWAFE--- -------
AlySkp01  LKAWDTEF MK-IDQAT LFELILAA NYLNIKNL LDLTCQTV -----ADM IKGKTPEE IRTTFNIK NDFTPEEE EE-VRREN QWAFE--- -------
```
