# Supplementary material for: Adaptive and degenerative evolution of the S-Phase Kinase-Associated Protein 1-Like family in Arabidopsis thaliana
Source: PeerJ. 2019 Apr 12;7:e6740. doi: 10.7717/peerj.6740 (PMC6463862; doi:10.7717/peerj.6740)
Supplement: Supplemental Information 5 [file peerj-07-6740-s005.pdf]

```
Query Protein: ASK1 IAHMVEDDCVDNGVPLPNVTSKILAKVIEYCKRHVEAAASKAEAVEGAA
 Consense Residues  I  M EDDC DNG+PLPNVTSKIL  VIEYCK+HV         VE
        Prediction  IKGMAEDDCADNGIPLPNVTSKILLLVIEYCKKHV---------VE---
                    aagaggggtggagacccagaaaacttgagttaacg         gg
  Target DNA: ASK6  tagtcaaagcaagtctcatcgatttttttaagaaat         ta
                    agtgaacttcttacttacgacgacaggcgtcggcc         tg

Query Protein: ASK1 TSDDDLKAWDADFMK-IDQATLFELILAANYLNIKNLLDLTCQTVADMI
 Consense Residues    ++DLK WDA+FMK  +Q+ LF++++AANYLNI++LLDLT QTVAD++
        Prediction  SKEEDLKKWDAEFMKKMEQSILFDVMMAANYLNIQSLLDLT!QTVADLL
                    aagggcaatgggtaaaagctactggaaggatcaacaccgcatcagggtc
  Target DNA: ASK6  gaaaataagacattaataactttatttccaatatagttatctactcatt
                    caaatagggctacggggaagtctttggtgttctcactttcatatcttgc
                                                             t

Query Protein: ASK1 KGKTPEEIRTTFNIKNDFTPEEEEEVRRENQWAFE
 Consense Residues   GKTP EIR+ FNI+NDFT E E E+R+ NQWAFE
        Prediction  SGKTPGEIRSYFNIENDFTAEGEAEIRKVNQWAFE
                    tgaacggactttaagagtagggggacagactgtg
  Target DNA: ASK6  cgaccgatgcatataaatccagacatgataagcta
                    acatacgtcacctcgctcaagaatgccggtagtta
```

Frame Shift mutation
