# Supplementary material for: Adaptive and degenerative evolution of the S-Phase Kinase-Associated Protein 1-Like family in Arabidopsis thaliana
Source: PeerJ. 2019 Apr 12;7:e6740. doi: 10.7717/peerj.6740 (PMC6463862; doi:10.7717/peerj.6740)
Supplement: Supplemental Information 6 [file peerj-07-6740-s006.pdf]

```
Query Protein: ASK1 MSAKKIVLKSSDGESFEVEEA--VALESQTIAHMVEDDCVDNGVPLPNV
 Consense Residues MS K IV+ SSDG++F+++EA  VA +S  I  M ED C DNG+PLPNV
        Prediction MSKKIIVVTSSDGDTFQIDEAR!VAFQSAMIKGMDEDKCADNGIPLPNV
                   ataaaaggaatgggatcagggcgggtctgaaagagggatggagacccag
Target DNA: AlySKP02 tcaattttcgcagactataacg tctaccttagtaaaagcaagtctcat
                   gaggacgggcctttttgccagc gacagggagtgtatgtcttacgtacc
                                            Frame Shift
                                            mutation

Query Protein: ASK1 TSKILAKVIEYCKRHVEAAASKAEAVEGAATSDDDLKAWDADFMK-IDQ
 Consense Residues TSKIL  VIEYCK+HV         VE    ++  LK WD +FMK  +Q
        Prediction TSKILLLVIEYCKKHV---------VEN--EEEEYLKKWDTEFMKKMEQ
                   aaaacttgagttaacg         gga  ggggtcaatgagtaaaagc
Target DNA: AlySKP02 cgattttttaagaaat         taa  aaaaataagacattaataa
                   acgccggttgtcggcc         cgc  aaaatcgggctacggggaa

Query Protein: ASK1 ATLFELILAANYLNIKNLLDLTCQTVADMIKGKTPEEIRTTFNIKNDFT
 Consense Residues + +F++++AANYLNI++L+DLTC+TVAD++ GKTPEEIR  F I+NDFT
        Prediction SIVFDVMMAANYLNIQSLIDLTCKTVADFLSGKTPEEIRAYFKIENDFT
                   tagtggaaggatcaacacagcataagggtctgaacggacgttaagagta
Target DNA: AlySKP02 ctttatttccaatatagttatcgactcattcgaccaatgcatataaatc
                   gtctttggtgttctcactttcgtatttttcaaataggtcaccgcgctta

Query Protein: ASK1 PEEEEEVRRENQWAFE
 Consense Residues PEEE E+ RENQWAFE
        Prediction PEEEAEILRENQWAFE
                   cggggacagactgtg
Target DNA: AlySKP02 caaacattgaaagcta
                   agaatgtcggtagttg
```
