# Supplementary material for: Adaptive and degenerative evolution of the S-Phase Kinase-Associated Protein 1-Like family in Arabidopsis thaliana
Source: PeerJ. 2019 Apr 12;7:e6740. doi: 10.7717/peerj.6740 (PMC6463862; doi:10.7717/peerj.6740)
Supplement: Supplemental Information 7 [file peerj-07-6740-s007.pdf]

```
Query Protein: ASK1     1 MSAKKIVLKSSDGESFEVEEAVALESQTIAHMVEDDCVDNGVPLPNVTS
Consense Residues         M+ K IVL SSDG+SF+++EAVA +S  I  M ED C DNG+PLPNVTS
Prediction                MAKKIIVLTSSDGDSFQIDEAVAFQSAMIKGMDEDKCADNGIPLPNVTS
                      501 agaaaagtaatgggttcaggggtctgaaagagggatggagacccagaa
Target DNA: AhaSkp11      tcaattttcgcagactataactctaccttagtaaaagcaagtctcatcg
                          ggggacgggcctttttgtcaggacagggagtgtatgtcttacttactac

Query Protein: ASK1    50 KILAKVIEYCKRHVEAAASKAEAVEGAATSDDDLKAWDADFMK-IDQAT
Consense Residues         KIL  VIEYCK+HV         VE     ++DLK WD +FMK  +Q+
Prediction                KILLLVIEYCKKHV---------VE--SNEEEDLKKWDTEFMKKMEQSI
                      648 aacttgagttaacg         gg  aaggggcaatgagtaaaagcta
Target DNA: AhaSkp11      atttttaagaaat          ta  gaaaaataagacattaataact
                          gccgggtgtcggcc         cg  ccaaatcgggctacggggaagt

Query Protein: ASK1    98 LFELILAANYLNIKNLLDLTCQTVADMIKGKTPEEIRTTFNIKNDFTPE
Consense Residues         +F++++AANYLNI++L+DLTCQTVAD++ GKTPEEIR  F I+ND+T E
Prediction                VFDVMMAANYLNIQSLIDLTCQTVADLLSGKTPEEIRAYFKIENDLT!E
                      762 gtggaaggatcaacacagcatcagggtctgaacggacgttaagagtacg
Target DNA: AhaSkp11      ttatttccaatatagttatcgactcattcgaccaatgcatataaatc a
                          ctttggtgttctcactttcttattttgcacataggtcatcgcgctaa g

Query Protein: ASK1   147 EEEEVRRENQWAFE
Consense Residues         EE ++R ENQWAFE
Prediction                EEAKIRMENQWAFE
                      907 gggaacagactgtg
Target DNA: AhaSkp11      aacatgtaaagcta
                          aatgtcggtagttg
```

Frame Shift mutation
